# An intact helical domain is required for Gα_14_ to stimulate phospholipase Cβ

**DOI:** 10.1186/s12900-015-0043-3

**Published:** 2015-09-16

**Authors:** Dawna HT Kwan, Ka M. Wong, Anthony SL Chan, Lisa Y. Yung, Yung H. Wong

**Affiliations:** Division of Life Science and the Biotechnology Research Institute, Hong Kong University of Science and Technology, Clear Water Bay, Kowloon, Hong Kong; State Key Laboratory of Molecular Neuroscience, Hong Kong University of Science and Technology, Clear Water Bay, Kowloon, Hong Kong

## Abstract

**Background:**

Stimulation of phospholipase Cβ (PLCβ) by the activated α-subunit of G_q_ (Gα_q_) constitutes a major signaling pathway for cellular regulation, and structural studies have recently revealed the molecular interactions between PLCβ and Gα_q_. Yet, most of the PLCβ-interacting residues identified on Gα_q_ are not unique to members of the Gα_q_ family. Molecular modeling predicts that the core PLCβ-interacting residues located on the switch regions of Gα_q_ are similarly positioned in Gα_z_ which does not stimulate PLCβ. Using wild-type and constitutively active chimeras constructed between Gα_z_ and Gα_14_, a member of the Gα_q_ family, we examined if the PLCβ-interacting residues identified in Gα_q_ are indeed essential.

**Results:**

Four chimeras with the core PLCβ-interacting residues composed of Gα_z_ sequences were capable of binding PLCβ2 and stimulating the formation of inositol trisphosphate. Surprisingly, all chimeras with a Gα_z_ N-terminal half failed to functionally associate with PLCβ2, despite the fact that many of them contained the core PLCβ-interacting residues from Gα_14_. Further analyses revealed that the non-PLCβ2 interacting chimeras were capable of interacting with other effector molecules such as adenylyl cyclase and tetratricopeptide repeat 1, indicating that they could adopt a GTP-bound active conformation.

**Conclusion:**

Collectively, our study suggests that the previously identified PLCβ-interacting residues are insufficient to ensure productive interaction of Gα_14_ with PLCβ, while an intact N-terminal half of Gα_14_ is apparently required for PLCβ interaction.

**Electronic supplementary material:**

The online version of this article (doi:10.1186/s12900-015-0043-3) contains supplementary material, which is available to authorized users.

## Background

The superfamily of G protein-coupled receptors (GPCRs) constitutes the largest group of cell surface detectors for extracellular signals. Upon ligand binding, conformational changes in the receptor trigger the activation of heterotrimeric G proteins, which consists of α, β, and γ subunits, and results in the activation of various downstream effectors [1, 2]. Gα proteins are classified into four main families named as Gα_s_, Gα_i_, Gα_q_, Gα_12/13_, while five Gβ and twelve Gγ isoforms have been identified to date. The diversity in G protein subunits allows disparate signaling pathways to be regulated by different receptors. Robust stimulation of phospholipase Cβ (PLCβ) is primarily mediated by GPCRs that utilize Gα_q_ proteins for signaling [3], thereby leading to diverse cellular responses that range from cell proliferation to differentiation. The four known isoforms of PLCβ (PLCβ1-4) [4] are all stimulated by GTP-bound Gα_q_ subunits [5], even though they are either enriched in the cytosol (PLCβ2 and PLCβ3) or at the plasma membrane (PLCβ1 and PLCβ4) [6]. PLCβ catalyzes the hydrolysis of phosphatidylinositol 4,5-bisphosphate (PIP_2_) into diacylglycerol and inositol 1,4,5-trisphosphate (IP_3_), and reciprocally acts as a GTPase activating protein (GAP) of Gα_q_ [7, 8]. Since there are several members within the Gα_q_ subfamily (Gα_q_, Gα_11_, Gα_14_, and Gα_15_/Gα_16_) and all are fully capable of stimulating PLCβ [5], numerous GPCRs employ the Gα_q_/PLCβ pathway to regulate different cellular functions. Moreover, the Gβγ complex released upon G protein activation can also stimulate PLCβ2 and PLCβ3 isoforms [9, 10]. Given the importance of the Gα_q_/PLCβ axis in cell growth [11], its dysregulation is expected to contribute to the pathophysiology of various diseases. Indeed, somatic mutations causing constitutive activation of Gα_q_ drive ~50 % of all uveal melanomas [12].

**Fig. 1:**
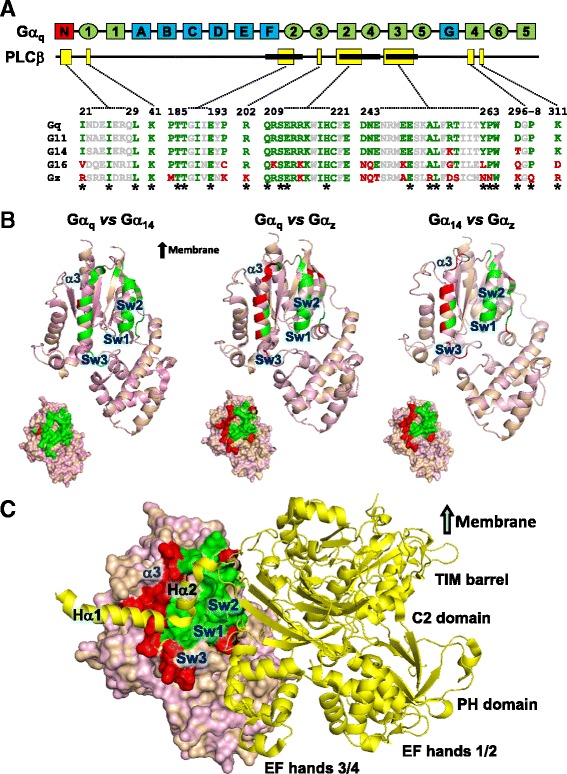
Alignment of PLCβ-interacting residues in Gα_q_ family and Gα_z_. **a** Schematic view of Gα_q_ divided into helical (light blue) and GTPase (light green) domains with α-helices and β-strands represented by rectangles and ovals, respectively. Interacting domains of Gα_q_ with PLCβ are indicated by yellow boxes below the Gα_q_ sequence; the three bold segments indicate the relative positions of the three switch regions (Sw1 to Sw3 from left to right). Sequence alignment of PLCβ-interacting domains in the Gα_q_ family as compared to that of Gα_z_; conserved (green) or divergent (red) PLCβ-interacting residues are interspersed by conserved residues which are not implicated in interaction with PLCβ (grey). Residues forming direct interactions with PLCβ3 as identified by Waldo *et al.* [13] are indicated by an asterisk. **b** Structural representation of Gα_q_, Gα_14_, and Gα_z_ alignments with switch regions (Sw1-3) and the α3 region. PLCβ3-interacting residues revealed in the sequence alignment are colored as indicated in ***A***. Space filling models are showing interacting surfaces. Structural models of Gα_14_ and Gα_z_ are generated based on Gα_q_-PLCβ3 (PDB code: 3OHM) using SWISS-MODEL [65, 66]. Structure alignments are carried out with PyMOL (The PyMOL Molecular Graphics System, Version 1.3 Schrödinger, LLC). **c** Complex of Gα_q_/Gα_z_-PLCβ3. The Gα_q_/Gα_z_ aligned model is represented as indicated in (B). PLCβ3 (yellow) is depicted as a cartoon ribbon, containing the helix-turn-helix segment (Hα1/Hα2), the N-terminal PH domain, four EF hands, the catalytic TIM barrel, and a C2 domain. The switch regions of Gα_q_ interact with PLCβ3 through an extended loop region between EF hands 3/4 and the region between the catalytic TIM barrel and C2 domain. The helix-turn-helix segment (Hα1/Hα2) at the C-terminus of PLCβ3 resides on the surface region formed by switch 2 and α3 of Gα_q_. The α_N_ helix of Gα proteins and carboxy-terminal (CT) domain of PLCβ3 are not included in the structural models

Despite intense efforts directed at understanding the interactions of Gα_q_ and PLCβ, the structure of a PLCβ-Gα_q_ complex has only been recently solved by molecular replacement manipulations using the crystal structures of PLCβ3 and an activated Gα_q_ [13]. The predicted structure of the PLCβ3-Gα_q_ complex has identified a series of discrete residues that form the interacting surfaces (Fig. 1a). According to the structural data, PLCβ3 binding occurs mainly at the switch regions of Gα_q_ (Fig. 1a and b). The switch I and II residues of Gα_q_ (green) interact with PLCβ3 through an extended loop region between EF hands 3/4, which is conserved in all PLCβ isoforms (Additional file 1: Figure S1), and the region between the catalytic TIM barrel and C2 domain of PLCβ3, providing an interface between PLCβ3 and Gα_q_ for interaction between a series of charged residue pairs. The highly conserved helix-turn-helix segment (Hα1/Hα2) at the C-terminus of the C2 domain of PLCβ3 resides on the surface region formed by switch II (α2-β4) and the α3 helix of Gα_q_ and allows the formation of various contacts with Gα_q_ in the large binding interface (Fig. 1c). More recently, discovery of the full-length structures of both PLCβ3 and Gα_q_ in complex has highlighted additional domains of PLCβ3 and Gα_q_ necessary for activation of lipid hydrolysis and protein interactions [14]. The crystallized full-length PLCβ3 contains a distal C-terminal domain (CTD) which is considered to be important for activation, membrane localization, and regulation by Gα_q_ proteins [15, 16]. The distal CTD adopts an orientation that makes direct contacts with the αN helix of Gα_q_ and most likely participates in binding with Gα proteins.

Regions of the Gα_q_ necessary for PLCβ interaction (namely, Ile^217^ to Lys^276^ which encompass the α2-β4-α3-β5 regions) have previously been identified by alanine-scanning mutagenesis [17] and they are appropriately positioned for the interaction with PLCβ3 (Fig. 1a). A total of 33 amino acids in small clusters along the β2 to α4 regions (except Ile^21^, Ile^25^, and Leu^29^, which lies in the αN helix, and Lys^41^ which lies on the β1 strand) of Gα_q_ are predicted to form intermolecular bonds with PLCβ [13, 14]. As expected, most interacting residues in the core regions are conserved in all other Gα_q_ members including Gα_11_, Gα_14_, and Gα_16_ (Fig. 1a). However, between 36 and 60 % of the identified PLCβ-interacting residues are also found in other Gα protein families, with members of the Gα_i_ family having the highest homology to Gα_q_ (Additional file 1: Figure S2) [18, 19]. For instance, Gα_z_ of the Gα_i_ subfamily exhibits close to 60 % identity with Gα_q_ in the core PLCβ-interacting regions (Fig. 1a). Such a high degree of identity is rather surprising especially when Gα_16_, which stimulates PLCβ, is only 74 % identical to Gα_q_ in the PLCβ-interacting regions (Fig. 1a). More interestingly, molecular modeling between Gα_q_ and Gα_z_ predicts that their differences in the PLCβ-interacting regions form a ring around a central core domain (Fig. 1b, space filled models), with most of the PLCβ contact points conserved between the two Gα subunits (Fig. 1c). This calls into question whether the residues identified by molecular replacement [13] are sufficient to provide PLCβ binding selectivity to Gα_q_ members. Although the identified residues are involved in the formation of the PLCβ3-Gα_q_ complex and are necessary in PLCβ3 activation as confirmed in IP_3_ studies [13], there may be additional regions in Gα_q_ members that determine selectivity for PLCβ.

It has been well established that constitutively active Gα_q_ subunits can efficiently stimulate PLCβ [20] but has no regulatory effect on adenylyl cyclase [21]. Early studies have employed chimeric Gα_q_/Gα_s_ and Gα_16_/Gα_z_ constructs to map the PLCβ and receptor interacting domains on the Gα_q_ and Gα_16_ subunits [17, 22]. It has not been demonstrated whether other Gα_q_ members such as Gα_14_ (with over 80 % sequence similarity with Gα_q_) utilize the same regions to interact with PLCβ. Likewise, it remains to be determined if other PLCβ isoforms such as PLCβ2 (with the highest resemblance to PLCβ3; Additional file 1: Figure S1) employ similar structural regions as PLCβ3 for coupling to active Gα_q_. By generating a series of Gα subunit chimeras and testing their abilities to functionally associate with PLCβ2 in HEK293 cells, we have demonstrated that an intact helical domain in the N-terminus of Gα_14_ is necessary for productive interaction with PLCβ.

## Results

### The PLCβ-interacting core regions of Gα_14_ are insufficient to stimulate PLCβ2

The PLCβ-interacting surfaces of Gα_q_ have been generally mapped to the β2-β3-α2-β4-α3 regions [13, 17], and these residues are mostly conserved among Gα_11_, Gα_14_, and Gα_16_ (Fig. 1a). Given that most of the PLCβ contact sites of Gα_q_ appear to be similarly present in Gα_i_ subunits (Additional file 1: Figure S2), it is possible to confer PLCβ-stimulating function upon a Gα_i_ subunit by incorporating Gα_q_-specific residues. This will also allow for the identification of any additional structural determinant on Gα_q_ which may specify interaction with PLCβ. In order to distinguish exogenous from endogenous Gα subunits, we have opted to use Gα_14_ as the backbone for constructing chimeras instead of Gα_q_ or Gα_11_. Unlike Gα_q/11_, Gα_14_ is not expressed in HEK293 cells [23] and it differs from Gα_q_ by only two amino acids (Lys^256^ and Thr^296^ in Gα_14_) in the PLCβ-interacting regions (Fig. 1a, b). To determine if Gα_14_ utilizes the same regions as Gα_q_ for PLCβ interaction, Gα_14_/Gα_z_ chimeras were made by swapping specific domains between Gα_14_ and Gα_z_. Gα_z_ was selected because it does not interact with PLCβ [24] or other Gα_q_ effectors such as TPR1 [25]. The chimeric approach is well suited for mapping functional domains on the Gα subunits because their tertiary structures highly resemble one another. Moreover, chimeras made with Gα_z_ and Gα_16_ proteins are structurally viable [22, 25].

**Fig. 2:**
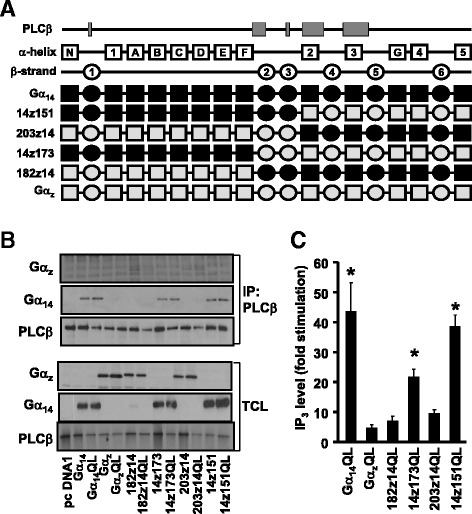
The putative PLCβ domain of Gα_14_ is not required for PLCβ interaction and activation. **a** Schematic representation of the 14z151, 203z14, 14z173, and 182z14 chimeras. Predicted secondary structures are illustrated as boxes (α helices) or circles (β strands) above the chimeras. Black areas represent human Gα_14_ sequence while those in grey signify the corresponding sequence of human Gα_z_. **b** HEK293 cells were co-transfected with PLCβ2 and the indicated Gα protein or chimeras. Cell lysates from the transfectants were immunoprecipitated by anti-PLCβ2 antiserum. The immunoprecipitates were immunoblotted with anti-Gα_14_, anti-Gα_z_ or anti-PLCβ2 antiserum. Aliquots of cell lysates were used to detect the expression levels of Gα_14_, Gα_z_ and PLCβ by Western blot analysis (TCL). Data shown represent one of three sets of immunoblots; two other sets yielded similar results. **c** HEK293 cells were transiently transfected with wild-type or constitutively active mutants (QL) of Gα protein or chimeras. Cells were then labelled and assayed for IP_3_ formation. Fold stimulations were calculated as the ratios of QL-induced to wild-type IP_3_ accumulations. Data represent the mean ± S.E.M. of three independent experiments, *n* = 3. *, IP_3_ production was significantly enhanced as compared to corresponding wild-type transfected cells; Dunnett *t* test, *p* < 0.05

Molecular modeling predicts that the differences between Gα_14_ and Gα_z_ in the PLCβ-interacting regions are distributed at the perimeters of the interacting surfaces, in much the same way as those of Gα_q_ versus Gα_z_ (Fig. 1b). Since most of the Gα_z_-specific sequences in the PLCβ-interacting domain reside in the α2-β4-α3 regions (Fig. 1a), we began by testing the importance of these regions by swapping the C-terminal half of Gα_z_ with Gα_14_ and assaying for the ability of the chimeras to interact with PLCβ2; PLCβ2 was chosen on the basis that it shares 76 % identity with PLCβ3 at the Gα_q_-interacting residues [13, 14]. The 14z151 chimera was constructed with the α2-β4-α3 regions together with the rest of the C-terminus of Gα_14_ (151 residues) replaced by the cognate sequence from Gα_z_; the mirror image of 14z151 was also constructed and named as 203z14 (Fig. 2a). Construction of the chimeras was guided by the predicted tertiary structure of the Gα subunits as well as by our previous experience in determining the receptor and effector interacting domains of various Gα subunits [22, 26–28]. A glutamine to leucine point mutation (QL) was introduced at Gln^205^ (equivalent to Gln^209^ in Gα_q_) to generate constitutively active mutants [29]. HEK293 cells were co-transfected with PLCβ2 in combination with pcDNA1, wild-type or constitutively active mutant of Gα_14_, Gα_z_, 14z151 or 203z14. As illustrated in Fig. 2b (upper panels), wild-type and constitutively active Gα_14_, but not those of Gα_z_, were successfully co-immunoprecipitated by anti-PLCβ2 antiserum and protein G sepharose. Chimera 203z14 did not interact with PLCβ2 despite having the α2-β4-α3 regions of Gα_14_ (Fig. 2b). This indicates that the other PLC-interacting regions of Gα_14_ (e.g., β2 and β3 regions) might be required for PLCβ2 interaction. More surprisingly, 14z151 was pulled down by anti-PLCβ2 even though its α2-β4-α3 regions were composed of Gα_z_ sequences.

To test the possibility that the β2-β3 regions are needed to maintain the overall structural integrity of the PLCβ-interacting surfaces, we further constructed a pair of chimeras with the junction extended forward to include the β2-β3 regions (Fig. 2a). Again, both the wild-type and constitutively active mutant of the chimera which harbored the β2-β3-α2-β4-α3 regions of Gα_14_ (182z14 and 182z14QL) failed to associate with PLCβ2, while their mirror images (14z173 and 14z173QL) co-immunoprecipitated with PLCβ2 (Fig. 2b). All of the chimeras and PLCβ2 were expressed at detectable and comparable levels in the total cell lysates (Fig. 2b, lower panels). These results suggest that the β2-β3-α2-β4-α3 regions, which are known to be important in Gα_q_ for PLCβ interaction, might not be sufficient for Gα_14_ to interact with PLCβ.

The co-immunoprecipitation results were subsequently confirmed by PLCβ functional assays. HEK293 cells were transfected with pcDNA1, Gα_14_, Gα_z_, the various chimeras or their constitutively active mutants and then subjected to IP_3_ accumulation assay. In agreement with previous reports [24, 30], expression of Gα_14_QL but not Gα_z_QL significantly stimulated IP_3_ formation (Fig. 2c). Both 14z151QL and 14z173QL also stimulated IP_3_ production whereas 182z14QL and 203z14QL failed to do so (Fig. 2c). None of the wild-type chimeras significantly affected IP_3_ production as compared to the vector controls (results not shown). Hence, these results demonstrate that the mere presence of the β2-β3-α2-β4-α3 regions of Gα_14_ does not necessarily confer upon the Gα subunit an ability to stimulate PLCβ. More interestingly, these regions can be functionally replaced by those from Gα_z_.

**Fig. 3:**
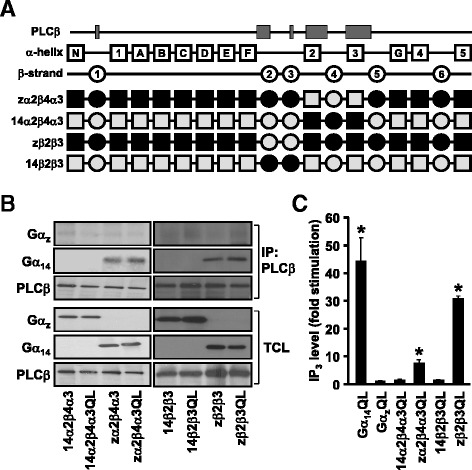
Role of β2-β3 and α2-β4-α3 regions of Gα_14_ in interaction and activation of PLCβ. **a** Schematic representation of zα2β4α3, 14α2β4α3, zβ2β3 and 14β2β3 chimeras. **b**, Cells were co-transfected with PLCβ2 and Gα protein or the indicated chimeras. Co-immunoprecipitation assays were performed and analyzed as in Fig. 2. Data shown represent one of three sets of immunoblots; two other sets yielded similar results. **c** HEK293 cells were transiently transfected with the wild-type or constitutively active mutants (QL) of Gα proteins or chimeras and then subjected to IP_3_ accumulation assay and analyzed as in Fig. 2. *, IP_3_ production was significantly enhanced as compared to corresponding wild-type transfected cells; Dunnett *t* test, *p* < 0.05

To test if the replacement of the PLC-interacting regions of Gα_14_ by cognate sequences from Gα_z_ can indeed support PLCβ activation, we swapped the β2-β3 or the α2-β4-α3 regions independently between the two Gα subunits (Fig. 3a). Among the various PLC-interacting regions, the α2 and α3 helices harbor most of residues that have been identified to form intermolecular bonds with PLCβ3 (Fig. 1a; [13]). Hence, substitution of the α2-β4-α3 regions in Gα_14_ with those of Gα_z_ might severely disrupt the ability of the resultant chimera (named as zα2β4α3) to interact with PLCβ. Although zα2β4α3 was co-immunoprecipitated by anti-PLCβ2 (Fig. 3b), its constitutively active mutant displayed a much weaker ability to induce IP_3_ formation as compared to Gα_14_QL (Fig. 3c). In contrast, chimera 14α2β4α3 (the mirror image of zα2β4α3) failed to associate or stimulate PLCβ2, suggesting that the α2-β4-α3 of Gα_14_ alone was insufficient to ensure PLCβ interaction. Likewise, we examined the role of the β2-β3 strands of Gα_14_ in PLCβ interaction. Chimera of Gα_14_ with the β2-β3 domain replaced by Gα_z_ (zβ2β3), and its mirror image (14β2β3), were constructed to determine if β2-β3 alone would affect PLCβ interaction with Gα_14_ (Fig. 3a). Our results showed that zβ2β3 remained capable of interacting with PLCβ and stimulating its activity (Fig. 3b, c), suggesting that the Gα_z_-specific residues in this region are sufficiently similar to those of Gα_14_ to allow productive interaction with PLCβ. On the other hand, 14β2β3 with most of the C-terminal and N-terminal of Gα_14_ replaced by Gα_z_, failed to interact with PLCβ2 or mediate IP_3_ production (Fig. 3b, c).

### The N-terminal helical domain of Gα_14_ is important for PLCβ interaction and activation

**Fig. 4:**
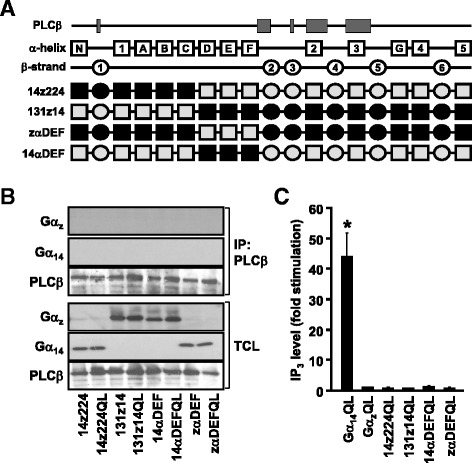
An intact N-terminal and helical domain are required for Gα_14_ mediated PLCβ interaction and activation. **a** Schematic representation of the 14z224, 131z14, 14αDEF and zαDEF chimeras. **b**, Cells were co-transfected with PLCβ2 and the indicated chimeras. Co-immunoprecipitation assays were performed and analyzed as in Fig. 2. Data shown represent one of three sets of immunoblots; two other sets yielded similar results. **c** HEK293 cells were transiently transfected with the wild-type or constitutively active mutants (QL) of Gα protein or the indicated chimeras and then subjected to IP_3_ accumulation assay and analyzed as in Fig. 2. *, IP_3_ production was significantly enhanced as compared to corresponding wild-type transfected cells; Dunnett *t* test, *p* < 0.05

The preceding results suggested that the N-terminal half (αN-αF) of Gα_14_ is seemingly important for PLCβ interaction and activation. Substituting the N-terminal of Gα_14_ from αN to αF with Gα_z_ completely abolished the ability of Gα_14_ to activate PLCβ even though the chimeras (182z14 and 203z14) can be successfully expressed (Fig. 2). To narrow down the residues in αN-αF which are involved in PLCβ activation, the N-terminal helical domain (αA-αF) of Gα_14_ was split into two halves and replaced by cognate sequences from Gα_z_ (Fig. 4a). The helical domain is essential for maintaining the overall structure of the Gα subunit and participates in effector regulation [31]. In order to minimize possible disruption to the Gα structure, the chimeras were designed to switch from Gα_14_ to Gα_z_ or vice versa at a position in the middle of the helical domain (Fig. 4a) where the residues of the two templates have high homology. Chimera 14z224 harboring the αN-αC of Gα_14_ was expressed efficiently but was unable to functionally associate with PLCβ (Fig. 4b, c). The mirror image of 14z224, chimera 131z14, also failed to interact with PLCβ or stimulate IP_3_ formation (Fig. 4b, c). Replacement of the second half of the helical domain (αD-αF) of Gα_14_ by Gα_z_ sequences, or vice versa, produced chimeras zαDEF and 14αDEF that neither interacted with PLCβ nor stimulated IP_3_ formation (Fig. 4b, c). It should be noted that chimeras 131z14 and zαDEF contained the putative PLCβ-interacting core domain (Fig. 4a).

**Fig. 5:**
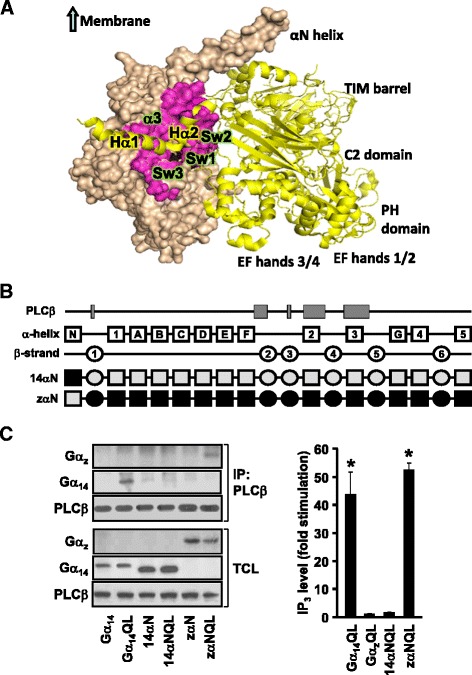
Role of the N-terminal helix (α_N_) in the Gα_q_-PLCβ3 complex. **a** The model of Gα_q_ (light orange) is shown as a space filling structure and contains the α_N_-helix and other regions as indicated. PLCβ3 (yellow) is depicted as a cartoon ribbon, containing the helix-turn-helix segment (Hα1/Hα2), the N-terminal PH domain, four EF hands, the catalytic TIM barrel, and a C2 domain. PLCβ3-interacting residues of Gα_q_ are colored in magenta. The carboxy-terminal (CT) domain of PLCβ3 is not included in the structural model. The structure of the α_N_-helix is generated by replacing the amino acid sequence of Gα_i_ (Gα_i_β_1_γ_2_, PDB code: 1GP2) with the Gα_q_ sequence. The final model is generated by alignment of Gα_q_-PLCβ3 (PDB code: 3OHM) and the modified heterotrimer Gα_q_β_1_γ_2_ using PyMOL (The PyMOL Molecular Graphics System, Version 1.3 Schrödinger, LLC). The orientation of the α_N_-helix represents the conformation in the heterotrimer and is not optimized for the Gα_q_-PLCβ3 complex. In this case, the α_N_-helix points towards the cell membrane and clashes with PLCβ3, but in fact may exist in a conformation which interacts with PLCβ3. **b** Schematic representation of 14αΝ and zαN chimeras. **c** Cells were co-transfected with PLCβ2 and Gα protein or the indicated chimeras. Co-immunoprecipitation assays were performed and analyzed as in Fig. 2. Data shown represent one of three sets of immunoblots; two other sets yielded similar results. For the IP_3_ accumulation assay, HEK293 cells were transiently transfected with the wild-type or constitutively active mutants (QL) of Gα proteins or chimeras and analyzed as in Fig. 2. *, IP_3_ production was significantly enhanced as compared to corresponding wild-type transfected cells; Dunnett *t* test, *p* < 0.05

The extreme N-terminus of the Gα subunit contains motifs for membrane localization and is thus often removed prior to crystallization [13]. By superimposing the N-terminal αN helix onto the crystal structure of Gα_q_, molecular modeling of the Gα_q_/PLCβ3 complex predicts that the αN helix may represent a contact site for PLCβ3 (Fig. 5a). Given that several chimeras (14z151, 14z173, zα2β4α3 and zβ2β3) were able to stimulate PLCβ activity despite having the PLCβ-interacting core region from Gα_z_, the provision of a Gα_14_ αN helix on a Gα_z_ backbone (14αN) might allow the resulting chimera to interact with PLCβ. However, chimera 14αNQL (Fig. 5b) did not stimulate IP_3_ formation whereas chimera zαNQL (Gα_14_ backbone with a Gα_z_αN helix) functionally interacted with PLCβ (Fig. 5c). Collectively, these results suggest that the αN helix is not a critical determinant in the recognition of PLCβ by Gα_14_.

### Non-PLCβ-interacting Gα_14_ chimeras can interact with other effectors

**Fig. 6:**
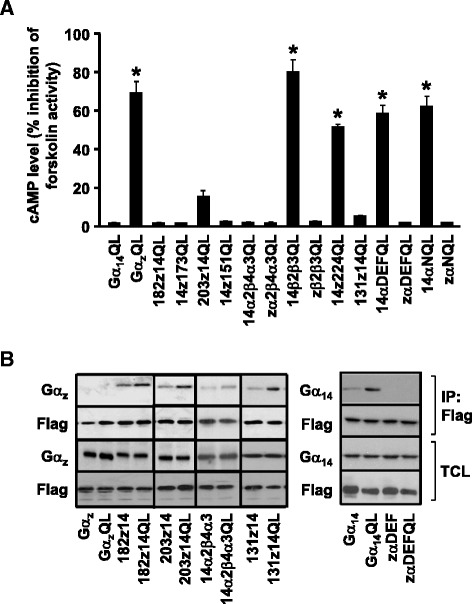
Ability of different chimeras to interact with AC and TPR1. **a** HEK293 cells were transiently transfected with the wild-type or constitutively active mutants of Gα protein and chimeras indicated in the figure. The transfectants were labelled with [^3^H]adenine (1 μCi/ml) in 1 % FBS/MEM. The labelled cells were treated with 50 μM of FSK for 30 min before subjected to cAMP accumulation assay. cAMP fold inhibition was calculated as the ratios of QL-induced to wild-type cAMP inhibition. Data represent the mean ± S.E.M. of three independent experiments, *n* = 3. *, cAMP accumulation was significantly inhibited as compared to corresponding wild-type transfected cells; Dunnett *t* test, *p* < 0.05. **b** HEK293 cells were transiently co-transfected with FLAG-TPR1 in combination with Gα proteins and the indicated chimeras. Cell lysates were immunoprecipitated by anti-FLAG affinity agarose gel. The immunoprecipitates were immunoblotted with anti-Gα_14_, anti-Gα_z_ or anti-FLAG antiserum. Crude lysates were used to examine the expression levels of Gα_14_, Gα_z,_ Gα_14_/Gα_z_ chimeras or FLAG-TPR1 by Western blot analysis. The immunoblots shown represent one of three sets of immunoblots; two other sets yielded similar results

Since nine chimeras (181z14, 203z14, 14α2β4α3, 14β2β3, 14z224, 131z14, zαDEF, 14αDEF, and 14αN) failed to interact with PLCβ despite clear evidence of expression, we sought to determine if these chimeric Gα subunits were in fact functional. Those chimeras harboring large segments of Gα_z_ sequence may behave like Gα_z_ and thus be capable of inhibiting adenylyl cyclase. The panel of chimeras was therefore subjected to cAMP accumulation assay. The ability of the constitutively active mutant of each chimera to inhibit forskolin-induced cAMP accumulation was compared to its corresponding wild-type chimera (Fig. 6a). Like Gα_z_QL, the constitutively active mutants of 14β2β3, 14z224, 14αDEF, and 14αN inhibited the forskolin response by 55-80 %, thereby confirming that these chimeras can adopt an active conformation. With four of the nine non-PLCβ-interacting chimeras demonstrating an ability to inhibit adenylyl cyclase, only five chimeras remained functionally unaccounted for.

Apart from being able to stimulate PLCβ by direct association [32, 33], Gα_14_ can also activate the Ras/ERK signaling pathway by interacting with TPR1 [34]. The Gα/TPR1 interaction is important for IKK and STAT3 phosphorylation via the Ras/ERK pathway [35, 36] and is apparently independent of PLCβ [25]. Since five chimeras (203z14, 182z14, 131z14, zαDEF, and 14α2β4α3) failed to exhibit any functional response in either IP_3_ or cAMP accumulation assays, we tested if these chimeras can associate with TPR1. HEK293 cells were co-transfected with an N-terminal Flag-tagged TPR1 (Flag-TPR1) and either the wild-type or the constitutively active mutant of Gα_14_, Gα_z_, or a chimera. Transfectants were subjected to co-immunoprecipitation using an anti-Flag affinity gel and protein G sepharose. The immunoprecipitates and cell lysates were then examined by western blot analysis using anti-Flag and either anti-Gα_14_ or anti-Gα_z_ antisera, depending on whether the N-terminus of the chimera is made up of Gα_14_ or Gα_z_ sequences. In agreement with previous studies [25, 34], neither Gα_z_ nor Gα_z_QL interacted with Flag-TPR1 whereas both Gα_14_ and Gα_14_QL co-immunoprecipitated with Flag-TPR1; noticeably more Gα_14_QL was associated with Flag-TPR1 (Fig. 6b). In contrast to Gα_z_, chimeras 203z14, 182z14, 131z14, and 14α2β4α3 were clearly detectable in the Flag-TPR1 immunoprecipitates (Fig. 6b); TPR1 interaction with 14α2β4α3 appeared to be weaker than the other chimeras. However, zαDEF could not be co-immunoprecipitated by Flag-TPR1 (Fig. 6b). Hence, only zαDEF did not exhibit any response in all of the functional assays. The ability of other chimeras to interact with Flag-TPR1 was similarly examined (Additional file 1: Figure S3) and the results are summarized in Table 1. Besides the inability of zαDEF to interact with Flag-TPR1, 14β2β3 and 14αN also exhibited negligible association with Flag-TPR1 but they were capable of coupling to adenylyl cyclase (Fig. 6a).

**Table 1:** Functional characterizations of the chimeras and their correlation with intact PLCβ or Gα_14_ helical domains

	PLCβ stimulation	Adenylyl cyclase inhibition	PLCβ interaction	TPR1 interaction	Intact PLCβ domain	Intact Gα_14_ helical domain
Gα_14_	Yes	No	Yes	Yes	Yes	Yes
203z14	No	No	No	Yes	Yes	No
14z151	Yes	No	Yes	Yes	No	Yes
182z14	No	No	No	Yes	Yes	No
14z173	Yes	No	Yes	Yes	No	Yes
14β2β3	No	Yes	No	No	No	No
zβ2β3	Yes	No	Yes	Yes	Yes	Yes
14z224	No	Yes	No	Yes	No	No
131z14	No	No	No	Yes	Yes	No
14αDEF	No	Yes	No	Yes	No	No
zαDEF	No	No	No	No	Yes	No
14α2β4α3	No	Yes	No	Yes	No	No
zα2β4α3	Yes	No	Yes	Yes	No	Yes
14αN	No	Yes	No	No	No	No
zαN	Yes	No	Yes	Yes	Yes	Yes
Gα_z_	No	Yes	No	No	No	No

Results of Gα proteins and chimeras in functional studies and co-immunoprecipitation assays are summarized. PLCβ stimulation was determined by measuring IP_3_ production by constitutively active (QL) chimeras as compared to their corresponding wild type activity (Figs. 2c, 3c, 4c, and 5c). The ability of QL-chimeras to inhibit adenylyl cyclase was determined in FSK-induced cAMP accumulation assays (Fig. 6a). Co-immunoprecipitation assays were performed using anti-PLCβ2 and anti-FLAG for the detection of PLCβ (Figs. 2b, 3b, 4b, and 5c) and TPR1 (Fig. 6b), respectively. Constructs containing an intact PLCβ binding domain (α2-β4-α3-β5 region) or an intact helical domain (αA-αF region) of Gα_14_ are also shown

Results obtained from the various assays are summarized in Table 1. Collectively, these results suggest that the core PLCβ-interacting regions are insufficient to ensure productive interaction with PLCβ and, more surprisingly, some of these regions can be functionally substituted by cognate residues from Gα_z_. Interestingly, an intact N-terminal helical domain (αA-αF) of Gα_14_ are seemingly important for Gα_14_-mediated PLCβ activation. Gα_14_ chimeras with αA-αF replaced entirely or in part by Gα_z_ can be expressed at a detectable level but failed to interact with PLCβ2 or stimulate IP_3_ production.

## Discussion

Structure and function correlations of members within the same protein family are often based on extensive analyses of a prototypical member. In the case of the Gα_q_ family, it is generally assumed that all members interact with the canonical effector PLCβ in much the same way as Gα_q_. The biochemical functions of Gα_q_ family members are almost indistinguishable [33, 37] except for their ability to recognize specific receptors [38]. Hence, it is rather surprising that the putative PLCβ-interacting domains identified from studies on Gα_q_ [13, 14, 17, 39] are simply insufficient to support efficient regulation of PLCβ by Gα_14_. Although detailed structural comparison between Gα_q_ and Gα_14_ is not feasible because of the lack of Gα_14_ structural data, the overall sequence similarity of over 80 % indicates a highly conserved three-dimensional structure shared by both proteins [18, 19]. Since the structural homology and the residues responsible for PLCβ interaction and activation are presumably conserved from Gα_q_ to Gα_14_, one would expect that Gα_14_ may utilize the same residues for PLCβ activation. It should also be noted that sequence variations in interacting residues of PLCβ2 and PLCβ3 may affect the ability of Gα_14_ to efficiently stimulate PLCβ2. In particular, conservative substitutions such as D973E and Q1066S in the distal CTD may have limited consequences for Gα_14_ binding, whereas more severe mutations in other interacting regions (E261S, Y855L, and R1062A) may significantly affect efficient activation by Gα proteins. Nonetheless, the substantial amount of conserved Gα_q_-interacting residues (76 %) in PLCβ2 should provide sufficient interacting regions for effector activation. However, Gα_14_/Gα_z_ chimeras lacking the putative PLCβ interacting domain (e.g., 14z151 and 14z173) are fully capable of interacting and stimulating PLCβ. Since these gain-of-function results do not correspond with current structural information on Gα_q_/PLCβ interaction [13, 14], it would appear that our understanding on how G proteins stimulate the PLCβ pathway is far from complete.

Gα_14_ and Gα_q_ resemble each other both structurally and biochemically. Both proteins are able to stimulate PLCβ2 and exhibit similar profiles of IP_3_ production [32]. Similar to other members of the Gα_q_ subfamily, Gα_14_ links a variety of G_q_-, G_s_-, and G_i_- coupled receptors to stimulate PLCβ3 [40–42]. In addition, palmitoylation of cysteine residues in the N termini of Gα_q_ and Gα_14_ is essential for membrane localization and efficient PLCβ activation [43, 44]. However, co-immunoprecipitation and PLCβ activation studies using Gα_14_/Gα_z_ chimeras suggested that an intact helical domain (αN-αF) of Gα_14_, but not the previously identified PLCβ3 interacting regions (α2-β4-α3-β5), is required for PLCβ interaction and activation. It should be noted that each Gα protein can be divided into the GTPase domain that comprises the PLCβ interacting regions and the helical domains composed of αA to αF helices [31]. There is increasing evidence to suggest that the helical domain participates in the activation and regulation of the Gα subunit [45]. For instance, the helical domain of Gα_16_ is known to bind GRK2 [46]. Substitution of the previously identified PLCβ3 interacting regions (α2-β4-α3-β5) of Gα_14_ by Gα_z_ is expected to abolish PLCβ interaction and activation. However, Gα_14_/Gα_z_ chimeras consisting of varying combinations of the interacting regions were able to interact and activate PLCβ. Most surprisingly, the 14z151QL chimera, consisting of an entire α2-β4-α3-β5 region of Gα_z_, was able to stimulate IP_3_ production to similar levels as Gα_14_, indicating that this region is not responsible for specifying interaction with PLCβ. The reduction in PLCβ activity of the 14z173QL chimera, consisting of an additional substitution of the adjacent β2-β3 region by Gα_z_, could be caused by its weaker binding with PLCβ, as lower protein levels of the chimera were observed in complex with PLCβ in the co-immunoprecipitation assay (Fig. 2b). Also, a GTPase domain consisting of an intact α2-β4-α3-β5 region of either Gα_14_ or Gα_z_ seems important for maximal activation of PLCβ. The zα2β4α3QL chimera disrupts this region and decreased IP_3_ production was observed without obvious binding defects as determined in the co-immunoprecipitation assay. In general, chimeras with substitutions in the GTPase domain of Gα_14_ showed limited binding defects but significant effects on IP_3_ production, which emphasizes the importance of the GTPase domain in PLCβ activation as compared to its less prominent role in protein binding.

Further co-immunoprecipitation and PLCβ activation studies using Gα_14_/Gα_z_ chimeras suggested that the helical domain of Gα_14_ is required for PLCβ activation. Replacing either half (amino acids 1–131, or 132–181) of the N-terminus of Gα_14_ by Gα_z_ disrupted the ability of the chimeras (14z224, 131z14, zαDEF, and 14αDEF) to interact with PLCβ, suggesting that an intact helical core is necessary for PLCβ binding. To date, the sites for effector binding have been mostly mapped to the GTPase domain [13, 14, 17, 39, 47, 48], while much less is known about the function of the helical domain. The helical domain is the most divergent among Gα subunits [49]. Early structural and sequence analyses on Gα predicted that the helical domain is involved in effector interaction and may act as a regulatory entry point for GPCRs and Gβγ subunits [49, 50]. Together with the GTPase domain, it forms the nucleotide binding pocket and regulates GDP/GTP exchange by altering the binding affinity of Gα and its substrate [51, 52]. It has been proposed to participate in G protein oligomerization [53] and in the transition between the inactive and active conformations of Gα [54]. Furthermore, the helical domain of Gα_s_ has been proposed to accelerate GTP hydrolysis by the GTPase domain, functioning as a GTPase-activating protein (GAP) [55]. A study using human/*Xenopus* chimeras of Gα_s_ subunit revealed that the helical domain of Gα_s_ is also important for the activation of adenylyl cyclase [56]. More recently, crystallization studies suggest that major displacement of the helical domain is required for receptor coupling [57], thereby proposing a role for the helical domain as the inhibitory barrier for receptor-dependent activation. Considering the potential functions of the helical domain, our present study supports the involvement of the helical domain in effector interaction and regulation.

According to recent structural data, the αN helix is an important structure of Gα subunits in many aspects [14]. It is required for the binding of Gβγ [58, 59] and GPCRs [27, 60, 61]. It is also the site of lipid modification which enables proper localization of the G proteins to the plasma membrane (reviewed in [62]). Truncation of the αN helix of Gα_q_ decreased Gα_q_-stimulated PLCβ3 activity without affecting the binding affinity (K_i_) with PLCβ3 [14]. Likewise, mutations in the hydrophobic surface of the distal CTD of PLCβ3, which is thought to form interactions with the αN helix of Gα_q_, did not decrease the binding affinity while IP_3_ production was still observed at considerable levels [14]. Consistently, our zαNQL chimera with the αN helix of Gα_14_ replaced by Gα_z_ did not show binding defects in co-immunoprecipitation assays and successfully induced IP_3_ production. Moreover, removal of the distal CTD domain of PLCβ3 has been shown to significantly inhibit IP_3_ production while only modestly affecting the binding affinity with Gα_q_ [14]. These results suggest that interaction between the distal CTD of PLCβ3 and the αN helix of Gα_q_ or Gα_14_ is important for maximal stimulation of PLCβ3 and plays a less prominent role in Gα protein binding.

Gα_z_ belongs to the Gα_i_ subfamily and is able to inhibit adenylyl cyclase (AC) activity and subsequent cAMP production by direct association with AC (Fig. 6). Accordingly, substitution of Gα_z_ by portions of Gα_14_ structure may affect the level of inhibition of cAMP production. Disruption of the GTPase domain of Gα_z_, as demonstrated by the 14α2β4α3 chimera, abolishes the ability of Gα_z_ to inhibit AC. The 14z224QL chimera significantly inhibited cAMP production as compared to 14z173QL, which lacks the αDEF region of Gα_z_ and was unable to inhibit AC. However, the 14αDEFQL chimera with an αDEF region of Gα_q_ significantly inhibited AC at comparable levels. Moreover, chimeras lacking the entire helical domain of Gα_z_ (14z151 and 14z173) were unable to inhibit AC. These results suggest that both an intact GTPase domain of Gα_z_, which agrees with previously reported literature [39, 48], and at least a portion of the helical domain of Gα_z_ is required for AC inhibition.

Gα_14_ and Gα_16_ belong to the same family and share high homology in terms of their amino acid sequence and signaling properties. Both Gα_14_ and Gα_16_ have been shown to activate Ras and downstream transcription factors such as NFκB and STAT3 through interaction with TPR1 [25, 34]. Previous studies using Gα_16_/Gα_z_ chimeras suggested that the β2 and β3 strands of Gα_16_ are important for the interaction with TPR1 but this is not necessarily the case for Gα_14_. Co-immunoprecipitation studies using Gα_14_/Gα_z_ chimeras (summarized in Table 1) suggest that TPR1 interacts with Gα_14_ and Gα_16_ through different structural regions. As demonstrated by the zαDEF chimera, the αDEF region of Gα_14_ seems necessary for interaction with TPR1. However, several chimeras lacking this region, including 182z14, 203z14, and 131z14, are able to interact with TPR1. The inability of the zαDEF chimera to interact with TPR1 could be caused by disruption of the helical domain resulting in instability of the protein structure. Disruption of the GTPase domain and substitution of the helical domain by Gα_z_, as demonstrated by the zα2β4α3 chimera, did not completely abolish TPR1 interaction. Moreover, the mirror image pairs 14z151 and 203z14, as well as 14z173 and 182z14, were able to interact with TPR1. These results indicate that the presence of either the α2-β4-α3 region of the GTPase domain or an intact helical domain of Gα_14_ is sufficient for TPR1 interaction.

## Conclusion

The present study has successfully used chimeric Gα_14_/Gα_z_ constructs to map critical regions for effector regulation and demonstrates the insufficiency of previous structural information in supporting efficient effector regulation by Gα proteins. Although the roles of the αN helix and helical domain of Gα subunits in G protein-mediated signal transduction have mostly been neglected, our results designate important roles for these domains of Gα_14_ in effector interaction and activation.

## Methods

### Reagents

The human cDNAs of Gα_14_, Gα_14_QL were obtained from Guthrie Research Institute (Sayre, PA, USA). Cell culture reagents, including LipofectAMINE PLUS reagents, and Lipofectamin 2000 were purchased from Invitrogen (Carlsbad, CA, USA). Anti-Gα_14_ targeting the N-terminal was obtained from Gramsch Laboratories (Schwabhausen, Germany). Anti-FLAG antibody and anti-FLAG affinity gel were from Sigma-Aldrich (St. Louis, MO, USA). Other antibodies were purchased from Cell Signaling Technology (Danvers, MA, USA). Protein G-agarose and dithiobis[succinimidylpropionate] (DSP) cross-linker were from Pierce Biotechnology (IL, USA). Osmonics nitrocellulose membrane and ECL kit were from Westborough (MA, USA) and Amersham (Piscataway, NJ, USA), respectively. Pertussis toxin (PTX) was obtained from List Biological Laboratories (Campbell, CA, USA), and octreotide (OCT) was from Sigma-Aldrich (St. Louis, MO, USA).

### Cell culture and co-immunoprecipitation

HEK293 cells were obtained from the American Type Culture Collection (CRL-1573, Rockville, MD). They were maintained in Eagle’s minimum essential medium at 5 % CO_2_, 37 °C with 10 % fetal bovine serum, 50 units/mL penicillin and 50 μg/mL streptomycin. For co-immunoprecipitation experiments, HEK293 cells were grown to 80 % confluency in 100 mm tissue culture plates and then co-transfected with 200 ng Gα and 200 ng FLAG-TPR1 cDNAs using 15 μL PLUS and LipofectAMINE reagents in Opti-MEM. Serum was replenished 3 h after transfection. Cross-linking was performed one day after transfection; transfected HEK293 cells were washed with PBS twice and then treated with 0.5 mM DSP in PBS for 15 min at room temperature. Cells were then washed again with PBS and maintained in quenching solution (50 mM glycine in PBS, pH 7.4) for 5 min. Subsequently, cells were lysed in ice-cold RIPA buffer (25 mM HEPES at pH 7.4, 0.1 % SDS, 1 % Nonidet P-40, 0.5 % sodium deoxycholate, 1 mM dithiothreitol, 200 μM Na_3_VO_4_, 4 μg/mL aprotinin,100 μM phenylmethylsulfonyl fluoride, and 2 μg/mL leupeptin). Cell lysates were gently rocked with an anti-Gα_14_ antiserum at 4 °C overnight, and then incubated in 30 μL protein G-agarose (50 % slurry) at 4 °C for 2 h. Alternatively, the cell lysates were incubated in 30 μL anti-FLAG affinity agarose gel (50 % slurry) at 4 °C overnight. Immunoprecipitates were washed with ice-cold RIPA buffer (400 μL) for four times, resuspended in 50 μl RIPA buffer and 10 μl 6× sample buffer and then boiled for 5 min. Gα_14_ and FLAG-TPR1 proteins in the immunoprecipitates were analyzed by Western blots.

### Construction of chimeras

Gα chimeras were constructed from cDNAs encoding human Gα_14_ and Gα_z_ by using polymerase chain reaction (PCR) techniques. The N-terminal 37, 131, 182 and 203 residues of Gα_14_ were substituted by the corresponding amino acids of Gα_z_ to generate zαN, 131z14, 182z14 and 203z14 chimeras, respectively. Primers were designed to produce two half-length fragments with overlapping regions; the forward fragment was generated with the antisense and T7 primers, whereas the backward fragment was made with the sense and reverse primers which target a BGH polyadenylation signal (BGH primers). The two half-products were then annealed together to generate a full-length fragment by another round of PCR using T7 and BGH primers. Mirror images of these constructs were generated analogously and were named 14αN, 14z224, 14z173 and 14z151 chimeras. PCR (30 cycles each with 94 °C for 60 s, 58 °C for 60 s and 72 °C for 90 s) was carried out using AccuPrime PCR mix. The 14β2β3 chimera was constructed using 182z14 as the DNA template for the forward half-product and 14z151 DNA template for the backward half-product. The zβ2β3 chimera was constructed using 14z173 as the DNA template for the forward half-product and 203z14 as the DNA template for the backward half-product. 14α2β4α3 was constructed using 203z14 as the template for the forward half product and Gα_z_ as the template for the backward half product. Its mirror image zα2β4α3 was constructed using 14z151 as the template for the forward half product and Gα_14_ for the backward half product. Finally, 14αDEF was constructed using 131z14 as the DNA template for the forward half-product and Gα_z_ as template for the backward half-product. Its mirror image zαDEF was constructed using 14z224 as the DNA template for the forward half-product and Gα_14_ as template for the backward half-product. Primers for chimera construction are listed in Table 2. All Gα chimeras were checked by restriction mapping and then subcloned into pcDNA3 at *Hind*III and *Xba*I sites. The constructs were confirmed by dideoxynucleotide sequencing using Applied Biosystem Big Dye Terminator v3.1 Cycle Sequencing Kits (Foster City, CA, USA).

**Table 2:** Primer sequences for constructing various Gα_14/z_ chimeras

Chimera	Templates	Primers
203z14	Gα_z_/Gα_14_	F: 5’- ATGGTGGACGTGGGG***GGCCAACGATCGGAA*** -3’
		R: 5’- ***TTCCGATCGTTGGCC***CCCCACGTCCACCAT -3’
14z151	Gα_14_/Gα_z_	F: 5’- ***ATGGTGGATGTTGGT***GGGCAGAGGTCAGAG -3’
		R: 5’- CTCTGACCTCTGCCC***ACCAACATCCACCAT*** -3’
182z14	Gα_z_/Gα_14_	F: 5’- CGCTCCCGGGACATG***ACCACCGGCATCATT*** -3’
		R: 5’- ***ATTGATGCCGGTGGT***CATGTCCCGGGAGCG -3’
14z173	Gα_14_/Gα_z_	F: 5’- ***CGCGTCCGAGTGCCC***ACCACGGGCATTGTG -3’
		R: 5’- CACAATGCCCGTGGT***GGGCACTCGGACGCG*** -3’
14β2β3	182z14/14z151	F: 5’- ***ATGGTGGATGTTGGT***GGGCAGAGGTCAGAG -3’
		R: 5’- CTCTGACCTCTGCCC***ACCAACATCCACCAT*** -3’
zβ2β3	14z173/203z14	F: 5’- ATGGTGGACGTGGGG***GGCCAACGATCGGAA*** -3’
		R: 5’- ***TTCCGATCGTTGGCC***CCCCACGTCCACCAT -3’
14z224	Gα_14_/Gα_z_	F: 5’- ***GCCATCAAGCAGCTC***TGGGCCGACCCAGGG -3’
		R: 5’- CCCTGGGTCGGCCCA***GAGCTGCTTGATGGC*** -3’
131z14	Gα_z_/Gα_14_	F: 5’- GTCATGCGACGGCTC***TGGCAAGATCCAGGC*** -3’
		R: 5’- ***GCCTGGATCTTGCCA***GAGCCGTCGCATGAC -3’
14αDEF	131z14/Gα_z_	F: 5’- ***CGCGTCCGAGTGCCC***ACCACGGGCATTGTG -3’
		R: 5’- CACAATGCCCGTGGT***GGGCACTCGGACGCG*** -3’
zαDEF	14z224/Gα_14_	F: 5’- CGCTCCCGGGACATG***ACCACCGGCATCATT*** -3’
		R: 5’- ***AATGATGCCGGTGGT***CATGTCCCGGGAGCG -3’
14α2β4α3	203z14/Gα_z_	F: 5’- ATGGTGGACGTGGGG***GGCCAACGATCGGAA*** -3’
		R: 5’- ***TTCCGATCGTTGGCC***CCCCACGTCCACCAT -3’
zα2β4α3	14z151/Gα_14_	F: 5’- ***ATGGTGGATGTTGGT***GGGCAGAGGTCAGAG -3’ R: 5’- CTCTGACCTCTGCCC***ACCAACATCCACCAT*** -3’
14αN	Gα_14_/Gα_z_	F: 5’- ***GGGCAGAGGTCAGAG***CGCGAAATCAAGCTG -3’ R: 5’- CAGCTTGATTTCGCG***CTCTGACCTCTGCCC*** -3’
zαN	Gα_z_/Gα_14_	F: 5’- AGCCAGCGGCAACGC***CGTGAGCTTAAGCTG*** -3’ R: 5’- ***CAGCTTAAGCTCACG***GCGTTGCCGCTGGCT -3’

Bold and italic nucleotides denote the Gα_14_-derived sequences. F, forward primer; R, reverse primer

### Inositol Phosphates (IP_3_) accumulation assay

HEK293 cells were seeded on a 12-well plate at 2 × 10^5^ cells/well one day prior to transfection. Cells were then transfected with 200 ng Gα using 2 μL PLUS and Lipofectamine reagents in Opti-MEM. On the next day, cells were labeled with inositol-free Dubecco’s modified Eagle’s medium (DMEM; 750 μL) containing 5 % FBS and 2.5 μCi/mL *myo*-[^3^H]inositol overnight. Labeled cells were washed twice with the inositol phosphates assay medium (DMEM buffered with 20 mM HEPES, pH 7.5 and 5 mM LiCl) and were incubated for 1 h at 37 °C. Reactions were stopped by replacing the assay medium with 750 μL ice-cold 20 mM formic acid and the lysates were kept in 4 °C for 30 min before the separation of [^3^H]IP from other labeled species by sequential ion-exchange chromatography as described previously [63].

### cAMP accumulation assay

HEK293 cells were labeled overnight with [^3^H]adenine (1 μCi/ml) in culture medium containing 1 % FBS. The labeled cells were rinsed once with 2 ml of assay medium (MEM containing 20 mM HEPES, pH 7.4) and incubated at 37 °C for 30 min with 1 ml of assay medium containing 1 mM 1-methyl-3-isobutylxanthine in the absence or presence of 50 μM forskolin. The cells were lysed with 1 ml 5 % trichloroacetic acid with 1 mM ATP to terminate the reaction and were stored at 4 °C for 1 h. Intracellular [^3^H]cAMP was isolated by sequential chromatography as described previously [64]. The level of [^3^H]cAMP was estimated by determining the ratios of [^3^H]cAMP to total [^3^H]ATP and [^3^H]ADP pools.

### Molecular modeling

Gα_q_ in a complex with PLCβ3 (PDB ID: 3OHM, [13]) was employed to illustrate the interaction between Gα and PLCβ, and for creating a molecular model of Gα_14_by homologous modeling using SWISS-MODEL [65, 66]. Visualization of various structures was accomplished using PyMOL (The PyMOL Molecular Graphics System, Version 1.3 Schrödinger, LLC).

### Western blotting analysis

Protein samples were resolved on 12 % SDS-polyacrylamide gels and transferred to Osmonics nitrocellulose membrane. Resolved proteins were detected by their specific primary antibodies and horseradish peroxidase-conjugated secondary antisera. The immunoblots were visualized by chemiluminescence with the ECL kit from Amersham, and the images detected in X-ray films were quantified by densitometric scanning using the Eagle Eye II still video system (Stratagene, La Jolla, CA, USA).

## Additional file

Additional file 1: Figure S1. Alignment of Gα_q_-interacting residues in the PLCβ family. **Figure S2.** Alignment of PLCβ-interacting residues in the Gα protein family. **Figure S3.** Interaction of Gα_14_/Gα_z_ chimeras with Flag-TPR1. (PPTX 306 kb)

## Abbreviations

AC: Adenylyl cyclase
ERK: Extracellular signal-regulated kinase
GPCRs: G protein-coupled receptors
HEK293: Human embryonic kidney 293
IP_3_: Inositol trisphosphate
MAPK: Mitogen-activated protein kinase
PLCβ: Phospholipase Cβ
TPR1: Tetratricopeptide repeat 1
