# Supplementary material for: An intact helical domain is required for Gα14 to stimulate phospholipase Cβ
Source: BMC Struct Biol. 2015 Sep 16;15:18. doi: 10.1186/s12900-015-0043-3 (PMC4573470; doi:10.1186/s12900-015-0043-3)
Supplement: Additional file 1: Figure S1. — Alignment of Gαq-interacting residues in the PLCβ family. Figure S2. Alignment of PLCβ-interacting residues in the Gα protein family. Figure S3. Interaction of Gα14/Gαz chimeras with Flag-TPR1. (PPTX 306 kb) [file 12900_2015_43_MOESM1_ESM.pptx]

## Slide 1
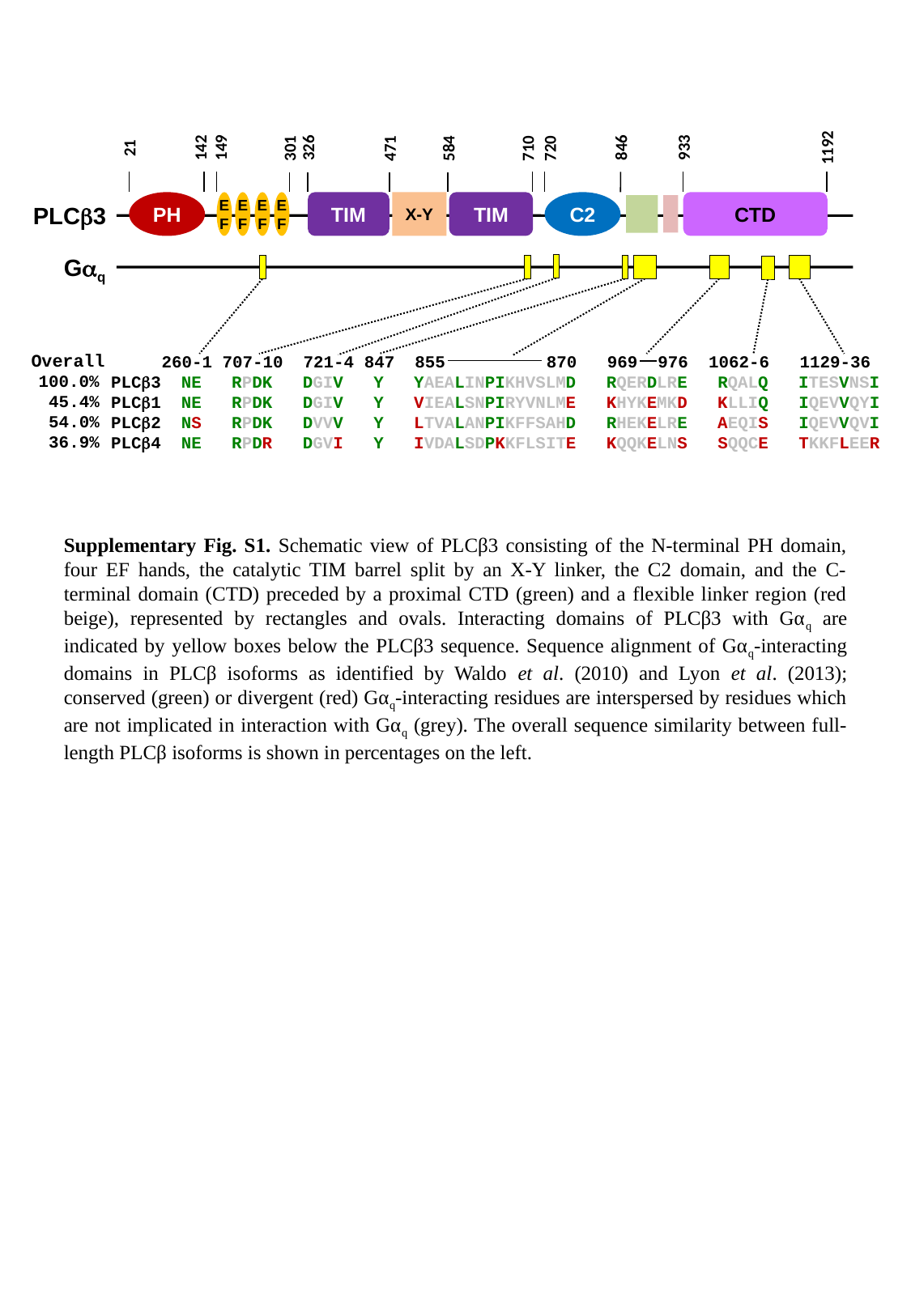

149
846
933
1192
142
326
21
301
471
584
710
720
PH
TIM
X-Y
TIM
C2
CTD
EF
EF
EF
EF
PLCb3
Gaq
Overall
 260-1 707-10 721-4 847 855 870 969 976 1062-6 1129-36
PLCb3 NE  RPDK DGIV Y YAEALINPIKHVSLMD RQERDLRE RQALQ ITESVNSI
PLCb1 NE  RPDK DGIV Y VIEALSNPIRYVNLME KHYKEMKD KLLIQ IQEVVQYI
PLCb2 NS  RPDK DVVV Y LTVALANPIKFFSAHD RHEKELRE AEQIS IQEVVQVI
PLCb4 NE   RPDR DGVI Y IVDALSDPKKFLSITE KQQKELNS SQQCE TKKFLEER
100.0%
45.4%
54.0%
36.9%
Supplementary Fig. S1. Schematic view of PLCβ3 consisting of the N-terminal PH domain, four EF hands, the catalytic TIM barrel split by an X-Y linker, the C2 domain, and the C-terminal domain (CTD) preceded by a proximal CTD (green) and a flexible linker region (red beige), represented by rectangles and ovals. Interacting domains of PLCβ3 with Gαq are indicated by yellow boxes below the PLCβ3 sequence. Sequence alignment of Gαq-interacting domains in PLCβ isoforms as identified by Waldo et al. (2010) and Lyon et al. (2013); conserved (green) or divergent (red) Gαq-interacting residues are interspersed by residues which are not implicated in interaction with Gαq (grey). The overall sequence similarity between full-length PLCβ isoforms is shown in percentages on the left.

## Slide 2
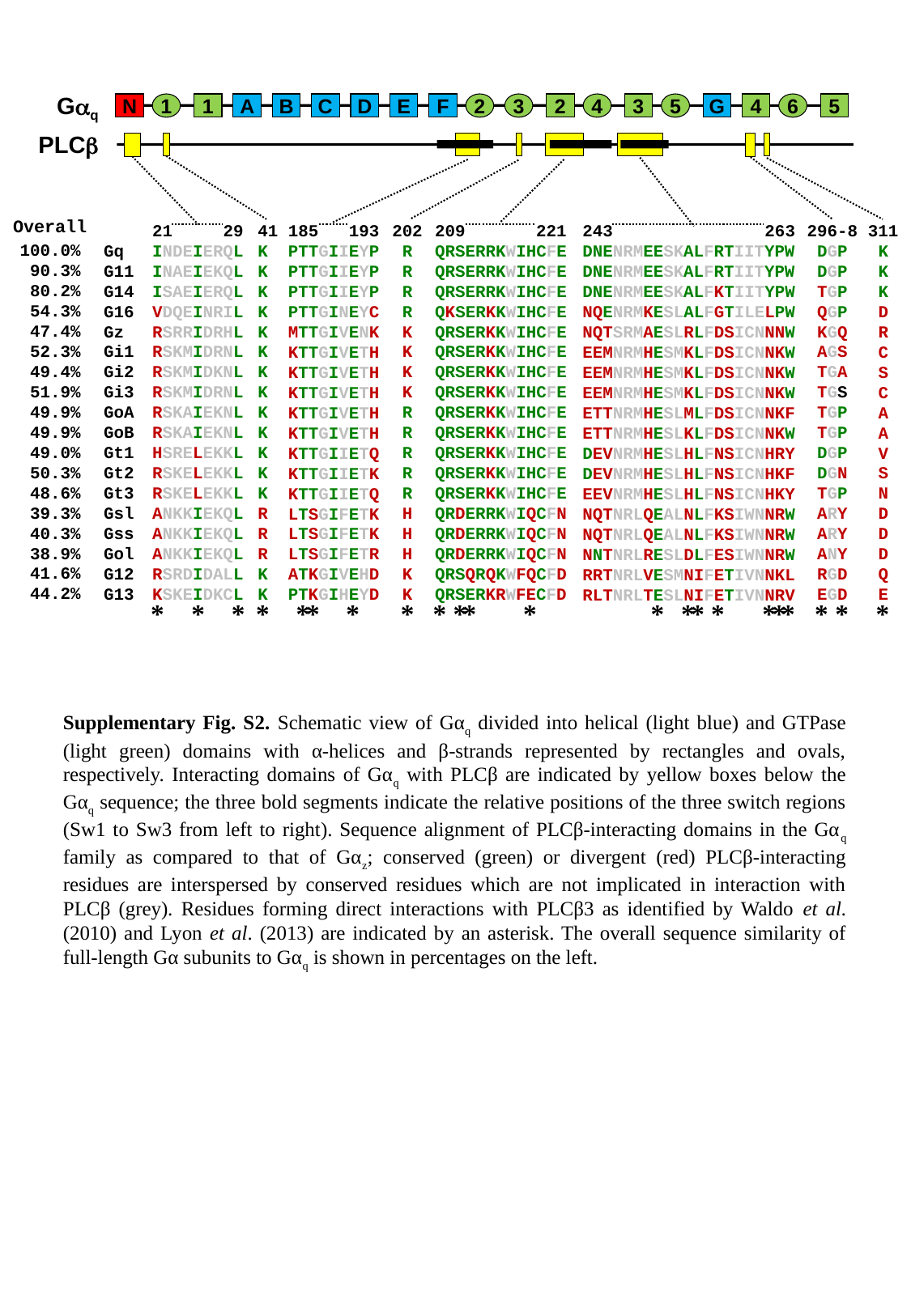

Gaq
N
1
1
A
B
C
D
E
F
2
3
2
4
3
5
G
4
6
5
PLCb
Overall
185 193
PTTGIIEYP
PTTGIIEYP
PTTGIIEYP
PTTGINEYC
MTTGIVENK
KTTGIVETH
KTTGIVETH
KTTGIVETH
KTTGIVETH
KTTGIVETH
KTTGIIETQ
KTTGIIETK
KTTGIIETQ
LTSGIFETK
LTSGIFETK
LTSGIFETR
ATKGIVEHD
PTKGIHEYD
243 263
DNENRMEESKALFRTIITYPW
DNENRMEESKALFRTIITYPW
DNENRMEESKALFKTIITYPW
NQENRMKESLALFGTILELPW
NQTSRMAESLRLFDSICNNNW
EEMNRMHESMKLFDSICNNKW
EEMNRMHESMKLFDSICNNKW
EEMNRMHESMKLFDSICNNKW
ETTNRMHESLMLFDSICNNKF
ETTNRMHESLKLFDSICNNKW
DEVNRMHESLHLFNSICNHRY
DEVNRMHESLHLFNSICNHKF
EEVNRMHESLHLFNSICNHKY
NQTNRLQEALNLFKSIWNNRW
NQTNRLQEALNLFKSIWNNRW
NNTNRLRESLDLFESIWNNRW
RRTNRLVESMNIFETIVNNKL
RLTNRLTESLNIFETIVNNRV
296-8
 DGP
 DGP
 TGP
 QGP
 KGQ
 AGS
 TGA
 TGS
 TGP
 TGP
 DGP
 DGN
 TGP
 ARY
 ARY
 ANY
 RGD
 EGD
Gq
G11
G14
G16
Gz
Gi1
Gi2
Gi3
GoA
GoB
Gt1
Gt2
Gt3
Gsl
Gss
Gol
G12
G13
21 29
INDEIERQL
INAEIEKQL
ISAEIERQL
VDQEINRIL
RSRRIDRHL
RSKMIDRNL
RSKMIDKNL
RSKMIDRNL
RSKAIEKNL
RSKAIEKNL
HSRELEKKL
RSKELEKKL
RSKELEKKL
ANKKIEKQL
ANKKIEKQL
ANKKIEKQL
RSRDIDALL
KSKEIDKCL
41
K
K
K
K
K
K
K
K
K
K
K
K
K
R
R
R
K
K
202
 R
 R
 R
 R
 K
 K
 K
 K
 R
 R
 R
 R
 R
 H
 H
 H
 K
 K
209 221
QRSERRKWIHCFE
QRSERRKWIHCFE
QRSERRKWIHCFE
QKSERKKWIHCFE
QRSERKKWIHCFE
QRSERKKWIHCFE
QRSERKKWIHCFE
QRSERKKWIHCFE
QRSERKKWIHCFE
QRSERKKWIHCFE
QRSERKKWIHCFE
QRSERKKWIHCFE
QRSERKKWIHCFE
QRDERRKWIQCFN
QRDERRKWIQCFN
QRDERRKWIQCFN
QRSQRQKWFQCFD
QRSERKRWFECFD
311
 K
 K
 K
 D
 R
 C
 S
 C
 A
 A
 V
 S
 N
 D
 D
 D
 Q
 E
100.0%
90.3%
80.2%
54.3%
47.4%
52.3%
49.4%
51.9%
49.9%
49.9%
49.0%
50.3%
48.6%
39.3%
40.3%
38.9%
41.6%
44.2%
*
*
*
*
*
*
*
*
*
*
*
*
*
*
*
*
*
*
*
*
*
*
Supplementary Fig. S2. Schematic view of Gαq divided into helical (light blue) and GTPase (light green) domains with α-helices and β-strands represented by rectangles and ovals, respectively. Interacting domains of Gαq with PLCβ are indicated by yellow boxes below the Gαq sequence; the three bold segments indicate the relative positions of the three switch regions (Sw1 to Sw3 from left to right). Sequence alignment of PLCβ-interacting domains in the Gαq family as compared to that of Gαz; conserved (green) or divergent (red) PLCβ-interacting residues are interspersed by conserved residues which are not implicated in interaction with PLCβ (grey). Residues forming direct interactions with PLCβ3 as identified by Waldo et al. (2010) and Lyon et al. (2013) are indicated by an asterisk. The overall sequence similarity of full-length Gα subunits to Gαq is shown in percentages on the left.

## Slide 3
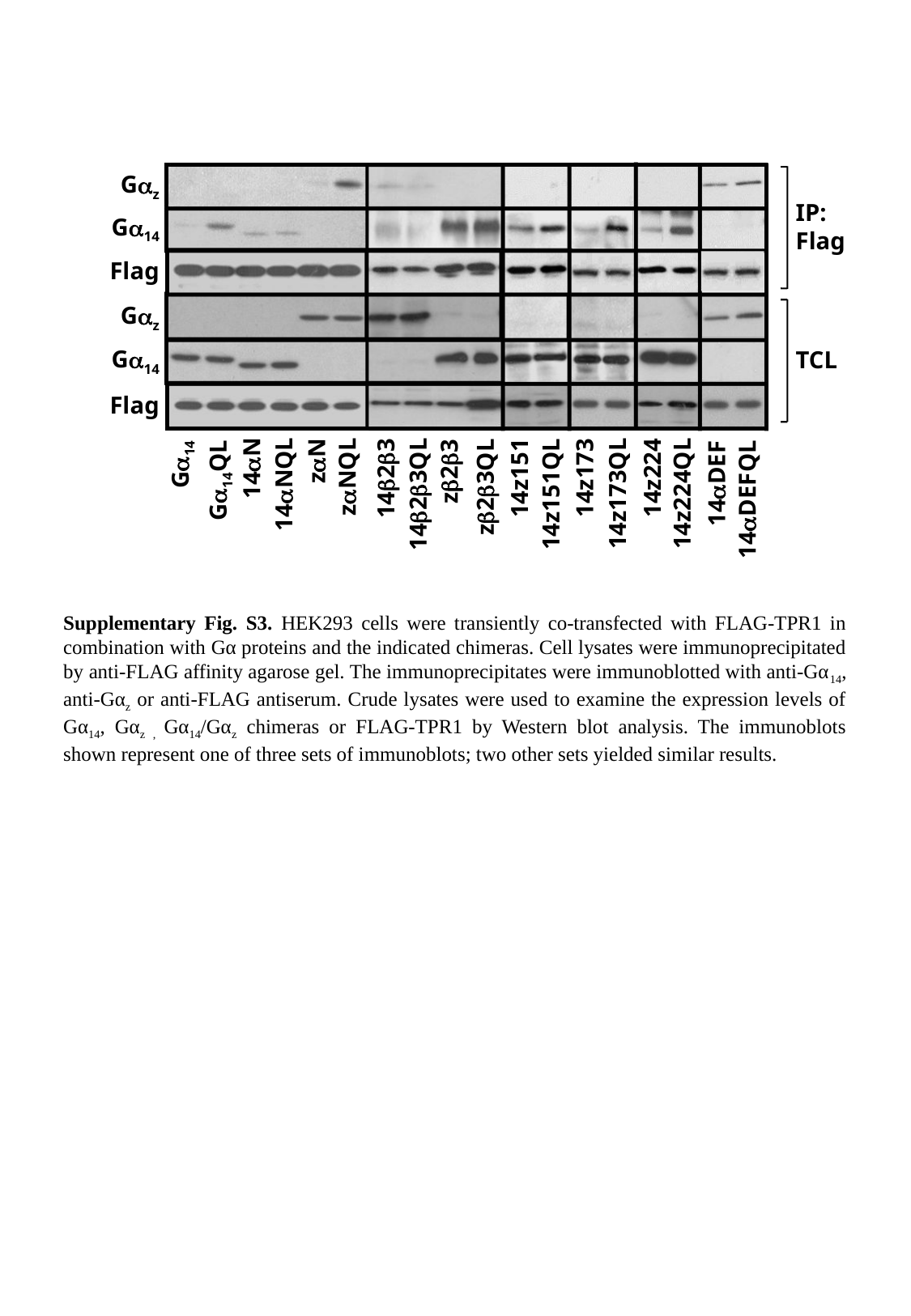

Gaz
IP: Flag
Ga14
Flag
Gaz
Ga14
TCL
Flag
zaN
Ga14
14aN
zb2b3
zaNQL
14z173
14z224
14b2b3
Ga14QL
14aDEF
14aNQL
zb2b3QL
14z173QL
14z224QL
14b2b3QL
14z151
14aDEFQL
14z151QL
Supplementary Fig. S3. HEK293 cells were transiently co-transfected with FLAG-TPR1 in combination with Gα proteins and the indicated chimeras. Cell lysates were immunoprecipitated by anti-FLAG affinity agarose gel. The immunoprecipitates were immunoblotted with anti-Gα14, anti-Gαz or anti-FLAG antiserum. Crude lysates were used to examine the expression levels of Gα14, Gαz , Gα14/Gαz chimeras or FLAG-TPR1 by Western blot analysis. The immunoblots shown represent one of three sets of immunoblots; two other sets yielded similar results.
